# Association between post-stroke cognitive impairment and gut microbiota in patients with ischemic stroke

**DOI:** 10.1038/s41598-025-03068-7

**Published:** 2025-05-29

**Authors:** Tsung-Min Jeng, Yi-Chen Hsieh, Po-Ya Chang, Yu-Ling Li, Sung-Chun Tang, Jiann-Shing Jeng, Chaur-Jong Hu, Hung-Yi Chiou

**Affiliations:** 1 Institute of Epidemiology and Preventive Medicine, College of Public Health, National Taiwan University, Taipei, Taiwan; 2 Ph.D. Program in Medical Neuroscience, College of Medical Science and Technology, Taipei Medical University, Taipei, Taiwan; 3 Department of Leisure Industry and Health Promotion, National Taipei University of Nursing and Health Sciences, Taipei, Taiwan; 4 Institute of Population Health Sciences, National Health Research Institutes, Miaoli County, Taiwan; 5 Department of Neurology, National Taiwan University Hospital, Taipei, Taiwan; 6 Department of Neurology, Shuang Ho Hospital, Taipei Medical University, New Taipei City, Taiwan; 7 Department of Neurology, School of Medicine, College of Medicine, Taipei Medical University, Taipei, Taiwan; 8 School of Public Health, College of Public Health, Taipei Medical University, Taipei, Taiwan

**Keywords:** Gut Microbiome, Inflammation, Ischemic stroke, Post-stroke cognitive impairment, Biomarkers, Neurology

## Abstract

More than half of stroke survivors have post-stroke cognitive impairment (PSCI). The role of gut microbiota, which can communicate with the brain through the gut-brain axis and affect inflammation, has been receiving increased attention. This cross-sectional study aimed to investigate the association of PSCI, gut microbiota, and inflammatory markers. Patients with first ischemic stroke and complete 3-month and 1-year follow-up data were included and divided into PSCI and non-PSCI groups according to the Montreal Cognitive Assessment (MoCA) score at the above time points. PSCI was defined as having a MoCA less than 23 at either 3 months or 1 year, or a decrease of more than 2 points at both time points. Gut microbiota was assessed by 16 S rRNA gene sequencing and Next Generation Sequencing analysis. The inflammatory markers included interleukins (ILs), eotaxin, G-CSF, TNF-α, IFNγ, sCD40L, and MCP-1. There were 95 ischemic stroke patients (mean age, 60.5 ± 12.1 years; male, 68.4%), including 30 with PSCI and 65 with non-PSCI. In gut microbiota analysis, the PSCI group had a higher abundance of Bacteroidaceae and Clostridiaceae, and the non-PSCI group had a higher abundance of *Prevotellaceae*, *Ruminococcaceae*, *Oscillibacter*, and *Faecalibacterium*. *Ruminococcaceae* family under the *Oscillospirales* order remains significantly different in the two groups in logistic regression model adjusting confounding variables (*p* = 0.044). In an analysis of inflammatory markers, the plasma levels of eotaxin (*p* = 0.041) and IL-12p40 (*p* = 0.031) were significantly higher in the PSCI group than those in the non-PSCI group, and the plasma level of eotaxin was significantly positively correlated with the amount of *Clostridiaceae* (rho = 0.389, *p* = 0.045). The study found that PSCI was associated with certain gut microbiota, and these gut microbiotas correlated with the pro-inflammatory marker eotaxin. This suggests that gut microbiota might play a role in the development of cognitive impairment after ischemic stroke.

## Introduction

Post-stroke cognitive impairment (PSCI) is highly prevalent and disabling after stroke^1–3^ According to one meta-analysis, the prevalence of post-stroke dementia at 1 year after stroke is 18.4%, rising to 20.4% when considering individuals with pre-existing dementia^4^ Another meta-analysis showed that 38% of stroke survivors had PSCI without dementia within the first year post-stroke^5^ Therefore, the combined prevalence of post-stroke cognitive impairment and dementia at 1 year after stroke exceeds half of all stroke survivors.

Factors determining the occurrence of PSCI include multiple aspects. These contain characteristics of the index stroke such as stroke lesions and types, non-modifiable risk factors such as age, genetic variation, and prior stroke, as well as modifiable risk factors including hypertension, diabetes, smoking, and education level. Additionally, factors related to brain health, such as brain atrophy, microbleeds, microinfarcts, white matter hyperintensity, and underlying neurodegenerative diseases, play significant roles^1,6–8^ Beyond these established risk factors, emerging research suggests a potential association between gut microbiota and post-stroke cognitive function.

The gut-brain axis involves various pathways, including the autonomic and enteric nervous system, the endocrine system, the hypothalamic-pituitary-adrenal axis, the immune system, the gut microbiota, and its metabolites^9–11^ This axis constitutes a bidirectional communication network connecting the gut and the brain, specifically involving the microbiome. Dysbiosis characterized by compositional and functional changes in the gut microbiota may contribute to stroke pathogenesis, progression, and outcomes^12,13^ Moreover, alterations in the gut-brain axis occur with aging, potentially influencing the gut microbiome, fostering age-related inflammation, and heightening the risk of stroke^14,15^ Besides, several important stroke risk factors, including type 2 diabetes, hypertension, obesity, and dietary habits such as high-fat and high-salt diets, are associated with the development of gut microbiome dysbiosis^16–18^.

Conversely, stroke-induced dysbiosis may result from various mechanisms. These include changes in diet and nutritional status,^19^ increased susceptibility to infections and subsequent antibiotics usage altering gut flora,^20,21^ impaired gastrointestinal function with decreased gastrointestinal motility, increased gastrointestinal bleeding, and altered microbiota composition^22,23^ Additionally, post-stroke immunosuppression,^24^ depression, and anxiety can weaken the immune system,^25^ further exacerbating dysbiosis.

Furthermore, systemic inflammation has emerged as a critical factor in developing PSCI. Recent studies showed the significance of inflammatory biomarkers, including C-reactive protein (CRP), and interleukins (IL) (IL-1β, IL-6, IL-8, and IL-10), and tumor necrosis factor-alpha (TNF-α), are associated with PSCI^26–28^ The present study aimed to investigate the association between PSCI, gut microbiota, and inflammatory markers in patients with ischemic stroke.

## Materials and methods

### Study design and study subjects

This is a cross-sectional study, and the study subjects were recruited from Taipei Medical University-Shuang Ho Hospital and National Taiwan University Hospital in Taipei, Taiwan, from January 2018 to December 2022. All participants were first-ever stroke patients and were confirmed by brain computed tomography (CT) or magnetic resonance imaging (MRI) within one week of stroke onset. According to Trial of Org 10,172 in Acute Stroke Treatment (TOAST), there are five ischemic stroke subtypes: large artery atherosclerosis, small vessel occlusion, cardioembolism, other determined etiology, and undetermined etiology^29^ Subjects were excluded if they had any of the following conditions: (1) post-stroke moderate-to-severe aphasia or severe dysarthria; (2) pre-stroke significant functional disability (modified Rankin scale [mRS] score > 1), dementia (Clinical Dementia Rating [CDR] scale score ≥ 1); (3) current infection or use of antibiotics within 2 weeks; (4) use of probiotics; or (5) unwilling to receive blood drawing or provide signed informed consent. Each patient underwent a comprehensive assessment, including a detailed medical history (focusing on stroke, vascular risk factors, co-morbidities), complete 3-month and 1-year follow-up of cognitive assessment, and fecal biospecimens collection 1 year after stroke.

This study received approval from the institutional review board of Taipei Medical University (N201805039) and National Taiwan University Hospital (201908008RINA). All methods were carried out following their respective guidelines and regulations. Written informed consent was obtained from the patients or their next of kin to ensure the absence of significant cognitive impairment.

## Cognitive evaluation and PSCI definition

The severity of cognitive impairment was measured by the Montreal Cognitive Assessment (MoCA) scale, which were performed by the study investigators and nurses who were blinded to the results of the blood testing. Cognitive and functional status assessments were conducted at 3 months and 1 year, respectively. The PSCI group was defined as having a MoCA score less than 23 at either 3 months or 1 year, or a decline of more than 2 points at both time points.

## Fecal sample extraction and 16 S rRNA sequencing

Fecal microbial DNA was purified and extracted using the QIAamp Fast DNA Stool Mini Kit (QIAGEN, Germany). Subsequently, microbial 16 S rRNA gene sequencing and next-generation sequencing analysis were performed using the Illumina MiSeq system. Targeted gene sequencing focused on the V3–V4 region of the bacterial 16 S rRNA gene, utilizing primers 341 F and 805 R. Following polymerase chain reaction (PCR), next-generation sequencing was conducted. Sequence data underwent processing using the R package DADA2 (v1.3.5) and were classified concerning the SILVA database (v128) for microbial taxonomy. The classification results were visualized using DECIPHER (v2.2.0) and Phangan (v2.2.0) software.

## Blood sample collection and cytokines assay

Blood samples of 10 mL were drawn and centrifuged at 2500×g for 15 min, then aliquoted into 1.5-mL tubes and stored at -80 °C until needed. The plasma samples were analyzed using a multiplex cytokine assay (Milliplex, MERCK) according to the manufacturer’s instructions and reported in pg/mL. We assessed 17 biomarkers from the multiplex assay: eotaxin, G-CSF (granulocyte colony-stimulating factor), IFNγ (interferon gamma), TNF (tumor necrosis factor)-α, sCD40L (soluble CD40 ligand), MCP (monocyte chemotactic protein)-1, IL-1α, IL-1β, IL-2, IL-4, IL-6, IL-8, IL-10, IL-12p40, IL-12p70, IL-13, and IL-17 A. The data were measured using a MAGPIX system, and xPONENT 4.2 software was used to analyze the data to determine the concentrations of various biomarkers.

### Statistical analysis

Continuous variables with a normal distribution were presented as the mean ± standard deviation or SEM. They were tested using a Student’s t-test between the groups. In contrast, variables with a non-normal distribution were shown as the median (interquartile range) and were examined by the Mann-Whitney U test. Categorical variables were expressed as frequency and percentages and evaluated using the χ2 test for comparisons.

Microbial communities were analyzed using phyloseq (v1.19.1) software. Alpha diversity was evaluated between the two groups using the Mann-Whitney U test (α = 0.05) to assess microbial richness. Beta diversity was determined by calculating UniFrac distances and using principal coordinates analysis to elucidate differences in microbial composition between the two groups. Statistical analysis employed Permutational Multivariate Analysis of Variance (PERMANOVA), also known as Adonis analysis (α = 0.05). LEfSe (Linear Discriminant Analysis (LDA) Effect Size) analysis was used to identify microbial species with notably distinct abundances between the two groups. Statistical testing was performed using Kruskal-Wallis and Mann-Whitney U tests (α = 0.05), with species having an LDA score greater than two considered to exhibit statistically significant differences. The graphical results were presented using GraPhlAn software. Additionally, logistic regression models were utilized to examine the association between microbiota and PSCI after adjusting for confounding factors. The correlation between microbiota and inflammatory biomarkers was analyzed using Spearman correlation methods. All statistical analyses were conducted using SAS ver. 9.4. (SAS Institute, Cary, NC) with two-sided probabilities. Statistical significance was defined as *p* < 0.05.

## Results

### Clinical characteristics

**Fig. 1:**
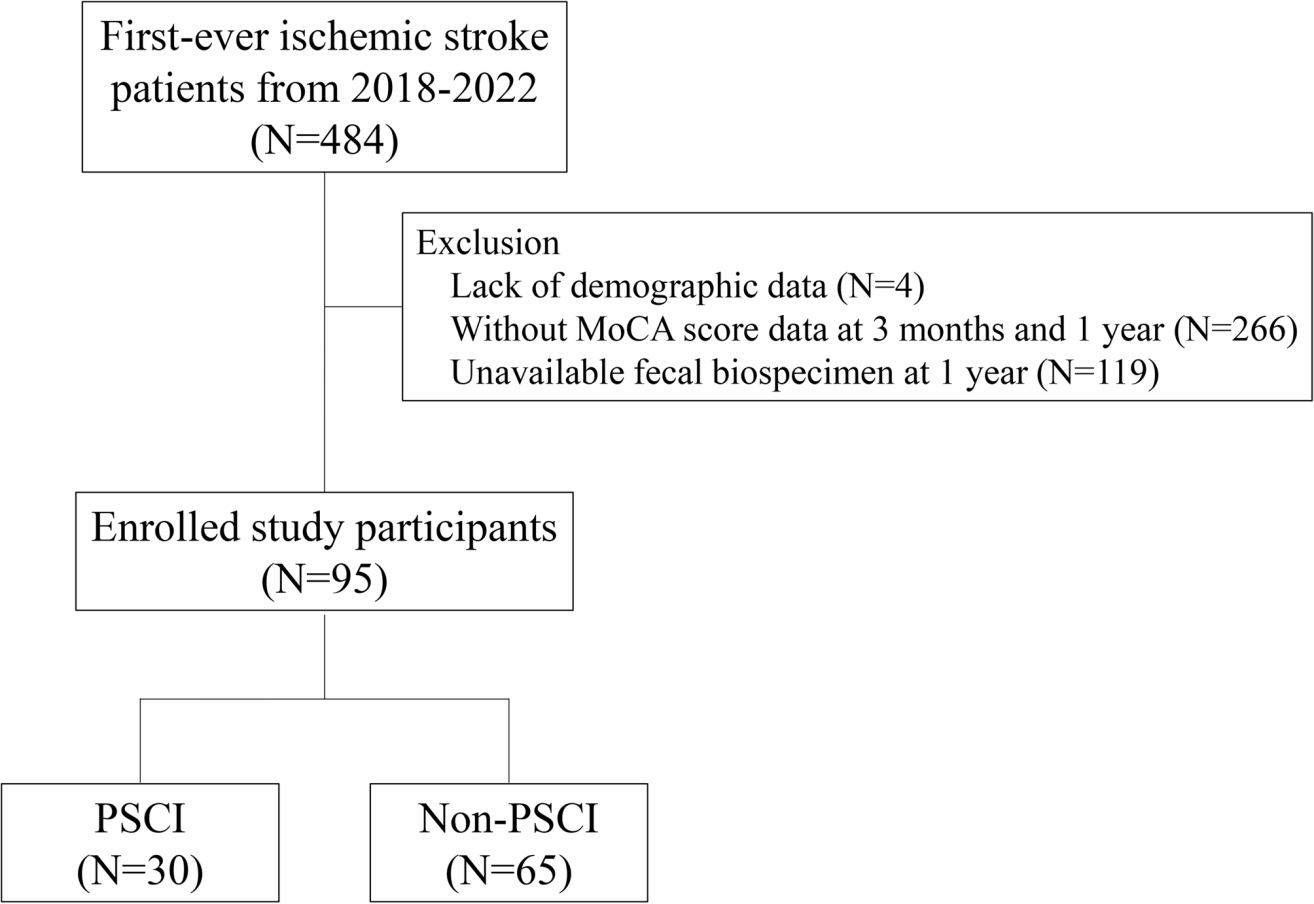
Flowchart of subject recruitment in this study. (PSCI, post-stroke cognitive impairment; non-PSCI, none of the post-stroke cognitive impairment; MoCA: Montreal Cognitive Assessment)

Our study enrolled 484 ischemic stroke patients between 2018 and 2022. Of these, 95 participants with complete demographic and clinical data, including 3-month and 1-year MoCA scores and available fecal samples, were included in our analyses (Fig. 1). The PSCI group comprised 30 participants, while the non-PSCI group included 65 participants. The demographics and clinical characteristics of all participants are shown in Table 1. Notably, individuals with PSCI were characterized by older age (65.8 ± 12.2 vs. 57.2 ± 10.8 years) and lower percentages of high school education completion compared to those without PSCI. However, there were no differences observed in terms of sex, ischemic stroke subtypes, comorbidities, and stroke severity between PSCI and non-PSCI patients. Moreover, patients with PSCI had higher MoCA scores more frequently than patients without PSCI at hospitalization, 3-month and 1-year post-stroke.

**Table 1:** Comparison of basic demographics and clinical characteristics between the PSCI and the non-PSCI groups.

	Total (*N* = 95)	PSCI (*N* = 30)	Non-PSCI (*N* = 65)	*p*
Age (year), _mean±SD_	60.5 ± 12.1	65.8 ± 12.2	57.2 ± 10.8	< 0.001
Male, _n(%)_	65 (68.4)	19 (63.3)	46 (70.8)	0.469
Education ≥ 12 years, _n(%)_	42 (45.6)	8 (28.6)	34 (53.1)	0.030
Martial status, _n(%)_				0.303
Married/Cohabitation	62 (67.4)	21 (75.0)	41 (64.1)	
Single	30 (32.6)	7 (25.0)	23 (35.9)	
Body mass index (kg/m^2^), _mean±SD_	26.0 ± 4.3	25.4 ± 4.0	26.4 ± 4.4	0.046
Ischemic stroke subtype, _n(%)_				0.199
Large artery atherosclerosis	20 (23.0)	9 (34.6)	11 (18.0)	
Small vessel occlusion	42 (48.3)	11 (42.3)	31 (50.8)	
Cardioembolism	9 (10.3)	1 (1.2)	8 (13.1)	
Other determined etiologies	4 (4.6)	0 (0.0)	4 (6.6)	
Undetermined etiology	12 (13.8)	5 (19.2)	7 (11.5)	
Risk factors, _n(%)_				
Hypertension	57 (66.3)	22 (78.6)	35 (60.3)	0.094
Diabetes mellitus	26 (31.7)	10 (37.0)	16 (29.1)	0.467
Heart disease	19 (23.8)	7 (28.0)	12 (21.8)	0.547
Dyslipidemia	59 (67.0)	19 (70.4)	40 (65.6)	0.659
Cigarette smoking	36 (42.4)	8 (30.8)	28 (47.5)	0.190
Alcohol drinking	23 (27.7)	6 (23.1)	17 (29.8)	0.524
NIHSS, _median (Q1−Q3)_				
Hospitalization	3 (1–5)	4 (2–8)	2 (1–4)	0.099
Leave	1 (0–3)	3 (1–4)	1 (0–2)	0.067
mRS, _median (Q1−Q3)_				
Hospitalization	1 (1–2)	2 (1–3)	1 (1–2)	0.118
3 months	1 (1–2)	2 (1–2)	1 (0–2)	0.090
1 year	1 (1–3)	2 (1–3)	1 (1–1)	0.637
MoCA, _median (Q1−Q3)_				
Hospitalization	25 (21–27)	20 (17–24)	26 (23–28)	0.005
3 months	26 (23–29)	22 (15–25)	28 (26–29)	< 0.001
1 year	26 (23–29)	19 (12–23)	28 (26–29)	< 0.001
Interval time (month), _median (Q1−Q3)_				
3 months - Onset	3.8 (2.8–4.9)	3.8 (2.8–7.7)	3.8 (9-4.6)	0.286
1 year - Onset	12.8 (11.7–14.4)	13.5 (11.5–15.7)	12.6 (11.7–13.5)	0.135

Values are mean ± standard deviation or median (interquartile range).

PSCI indicates post-stroke cognitive impairment; Non-PSCI indicates none of the post-stroke cognitive impairment; NIHSS, National Institute of Health Stroke Scale; mRS, modified Rankin Scale; MoCA, Montreal Cognitive Assessment.

## Microbial diversity and structure between the groups

**Fig. 2:**
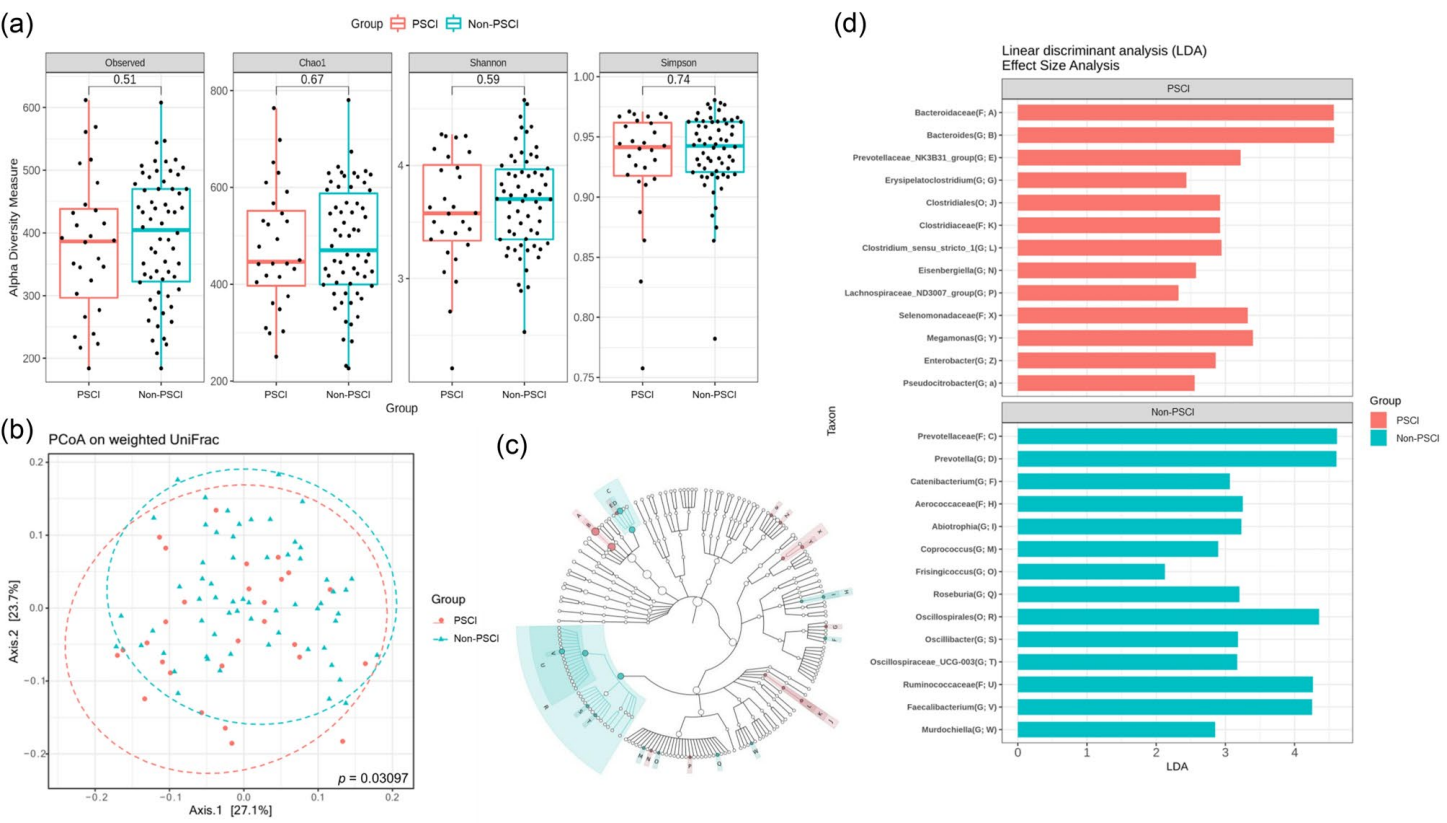
Microbial diversity in the PSCI and non-PSCI groups. (**a**) Alpha diversity compares the richness and diversity of the gut microbiota using observed, Chao1, Shannon, and Simpson indices. Statistical *p*-value using the Mann-Whitney U test (α = 0.05); (**b**) Beta diversity used the principal coordinate analysis based on the weighted UniFrac indices to elucidate differences in microbial composition between the two groups. Statistical analysis employed Adonis analysis (α = 0.05); (**c**–**d**) Significant bacterial differences between the two groups were determined using Linear discriminant analysis (LDA) Effect Size (LEfSe analysis). Red represents more bacteria abundance in the PSCI group, and blue represents more bacteria abundance in the non-PSCI group. (PSCI = Red, Non-PSCI = Blue; PSCI, post-stroke cognitive impairment; non-PSCI, none of the post-stroke cognitive impairment)

Alpha diversity analysis was used to compare the richness and diversity of the gut microbiota. In general, the richness and diversity were relatively lower in the PSCI group than in the non-PSCI group. The richness was represented as Observed (*p* = 0.51), Chao1 (*p* = 0.67), Shanon (*p* = 0.59), and Simpson (*p* = 0.74) indices were not significantly different between the two groups. The diversity of gut microbiota, represented by the Shannon and Simpson indices, also showed no difference between the PSCI and non-PSCI groups (Fig. 2A). Beta diversity analysis was used to compare the similarity of the bacterial community structures between the two groups, and it was evaluated using principal coordinates analysis. The weighted UniFrac indices were both found significant between the two groups (Adonis *p* = 0.031) (Fig. 2B), indicating that these two groups had dissimilar microbiota structures.

### Abundance of microbiota between the groups

**Fig. 3:**
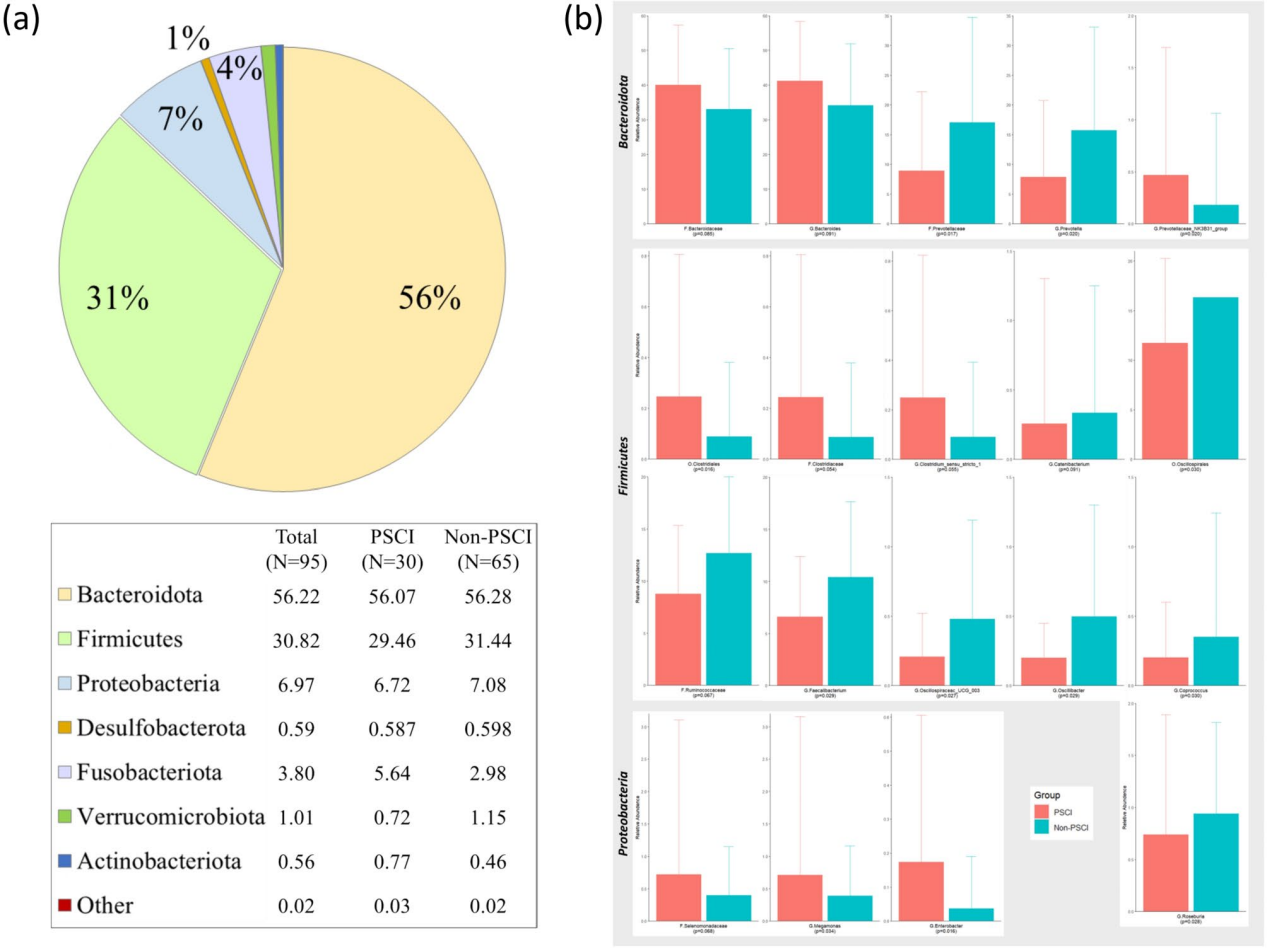
Microbiome abundance in the PSCI and non-PSCI groups. (**a**) Phylum relative abundance in the total samples, PSCI group, and non-PSCI group; (**b**) Different level relative abundance in the PSCI and non-PSCI groups. (PSCI = Red, Non-PSCI = Blue; PSCI, post-stroke cognitive impairment; non-PSCI, none of the post-stroke cognitive impairment; O, order; F, family; G, genus)

**Fig. 4:**
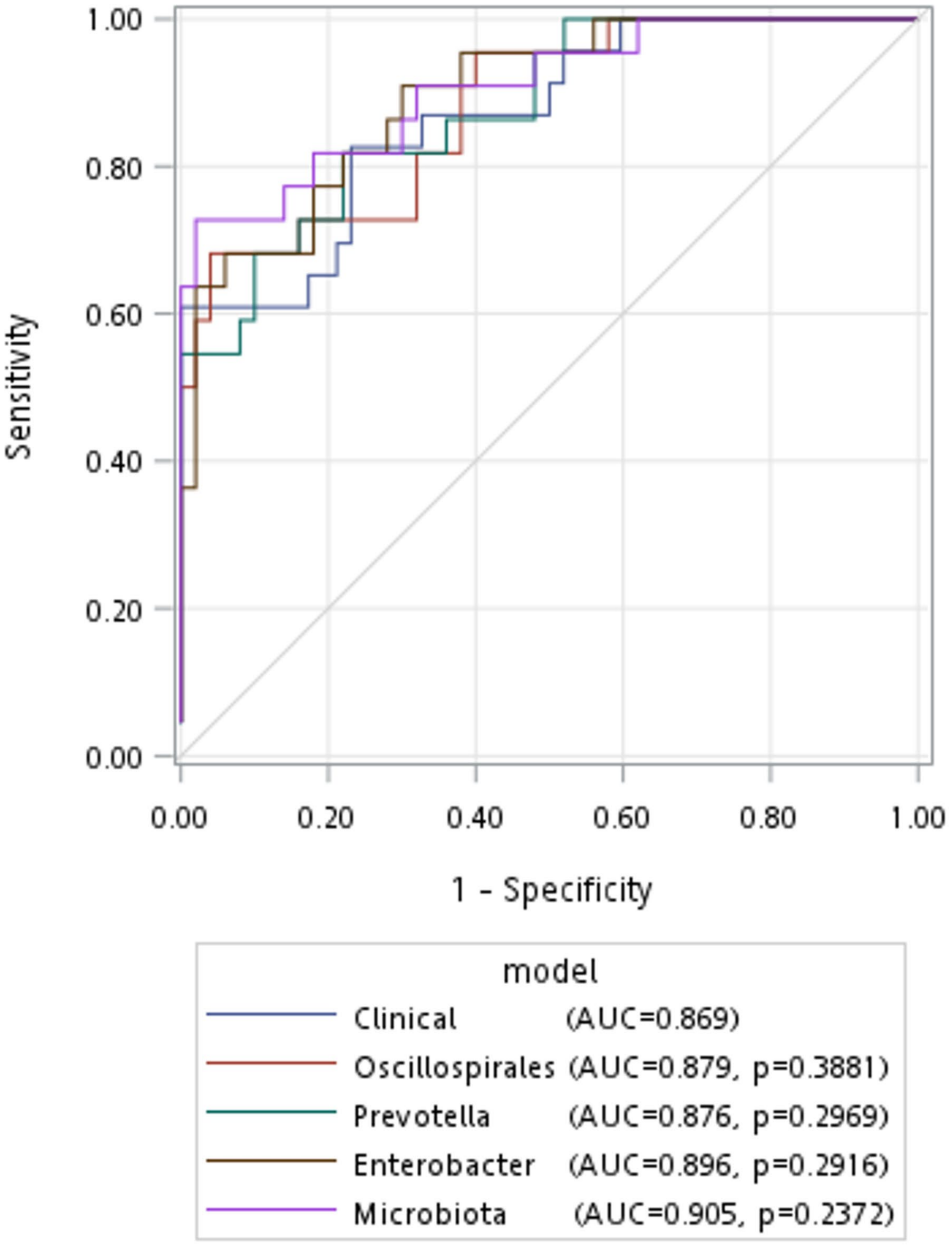
Comparison of the PSCI outcome prediction with ROC curves. Blue, clinical variables (age, gender, educational status, BMI, and mRS); Red, *Oscillospirales*; Green, *Prevotella*; Brown, *Enterobacter*; Purple, Microbiota = Oscillospirales + Prevotella + Enterobacter; The *p*-value is a comparison with clinical variables line. (PSCI, post-stroke cognitive impairment; non-PSCI, none of the post-stroke cognitive impairment; ROC, receiver operating characteristic; AUC, area under the ROC curve)

Among all patients, the leading phylum of gut microbiota were *Bacteroidota* (56.22%), *Firmicutes* (30.82%), *Proteobacteria* (6.97%), *Fusobacteriota* (3.80%), and *Verrucomicrobiota* (1.01%) (Fig. 3A). However, no significant difference was observed between the two groups at the phylum level. Figure 2C and D used LEfSe analysis to identify significant differences in microbiota between the two groups. At the family level, the PSCI group exhibited an increased abundance of *Bacteriodaceae*, *Clostridaceae*, and *Selenomonadeaceae*, while displaying a decreased abundance of *Prevotellaceae* and *Ruminococcaceae* compared with the non-PSCI group. At the genus level, the PSCI group showed elevated abundance of *Bacteroides*, *Clostridium_sensu_stricto_1*, and *Megamonas*, and decreased abundance of *Prevotella*, *Faecalibacterium*, and *Oscillibacter*. Based on the microbiota selected in the LEfSe analysis, we directly compared their relative abundance using the Mann-Whitney U test to determine if there was a statistically significant difference. Although some did not reach statistical significance due to differences in statistical methods, the results were generally consistent (Table 2; Fig. 3). The logistic regression model was conducted to ensure the robustness of outcomes after adjusting confounding variables using microbiota with a relative abundance of more than 0.1%. Table 3 shows that the *Oscillospirales* order, *Ruminococcaceae* family, *Prevotellaceae* family, *Prevotella* genus, and *Faecalibacterium* genus significantly differed in the two groups in the crude regression model (*p* = 0.020, 0.039, 0.021, 0.040, 0.020, respectively). After adjusting for the traditional demographic factors of age, gender, and educational status in model II, the *Oscillospirales* order, *Ruminococcaceae* family, *Faecalibacterium* genus, and *Enterobacter* genus significantly differed in the two groups (*p* = 0.046, 0.043, 0.017, 0.040, respectively). However, the *Prevotellaceae* family was non-significant between the two groups after being adjusted for confounding variables. The *Ruminococcaceae* family under the *Oscillospirales* order, remains significantly different in the two groups in full models with additional adjustments for BMI and mRS (*p* = 0.044). The significant microbiome groups identified in the regression models were chosen to predict outcomes using ROC curves (Fig. 4). When we use clinical variables (age, gender, educational status, BMI, and hospitalization mRS) to predict cognitive impairment, the AUC is 0.869. The addition of *Oscillospirales*, *Prevotella*, and *Enterobacter* could all slightly improve the AUC compared to clinical variables alone (0.873, 0.876, 0.893 respectively), although there was no statistical significance.

**Table 2:** Association between the expression of gut Microbiome and post-stroke cognitive impairment.

	Increased abundance in the PSCI group				Decreased abundance in the PSCI group			
Level	Microbiota	PSCI (*n* = 30)	Non-PSCI (*n* = 65)	*p*	Microbiota	PSCI (*n* = 30)	Non-PSCI (*n* = 65)	*p*
Order	*Clostridiales*	0.25 ± 0.56	0.09 ± 0.29	0.016	*Oscilliospirales*	11.73 ± 8.51	16.32 ± 8.15	0.030
Family	*Bacteroidaceae*	40.02 ± 17.26	33.02 ± 17.52	0.085	*Prevotellaceae*	8.92 ± 13.30	17.03 ± 17.69	0.017
	*Clostridiaceae*	0.24 ± 0.56	0.09 ± 0.29	0.054	*Aerococcaceae*	0.0001 ± 0.0001	0.0004 ± 0.0016	0.067
	*Selenomonadaceae*	0.72 ± 2.38	0.40 ± 0.75	0.068	*Ruminococcaceae*	8.77 ± 6.56	12.69 ± 7.31	0.081
Genus	*Bacteroides*	41.17 ± 17.17	34.16 ± 17.75	0.091	*Prevotella*	7.86 ± 12.89	15.74 ± 17.34	0.020
	*Prevotellaceae NK3B31 group*	0.47 ± 1.22	0.18 ± 0.18	0.020	*Catenibacterium*	0.26 ± 1.05	0.33 ± 0.92	0.091
	*Erysipelatoclostridium*	0.08 ± 0.22	0.03 ± 0.11	0.082	*Abiotrophia*	0.0001 ± 0.0001	0.0004 ± 0.0020	0.067
	*Clostridium_sensu_stricto_1*	0.25 ± 0.57	0.09 ± 0.30	0.055	*Coprococcus*	0.20 ± 0.40	0.35 ± 0.89	0.030
	*Eisenbergiella*	0.08 ± 0.32	0.01 ± 0.01	0.034	*Frisingicoccus*	0.01 ± 0.01	0.02 ± 0.14	0.001
	*Megamonas*	0.71 ± 2.44	0.39 ± 0.77	0.034	*Roseburia*	0.74 ± 1.15	0.94 ± 0.88	0.028
	*Enterobacter*	0.17 ± 0.43	0.04 ± 0.15	0.016	*Oscillibacter*	0.20 ± 0.25	0.50 ± 0.80	0.029
	*Pseudocitrobacter*	0.0004 ± 0.001	0.0002 ± 0.001	0.060	*Oscillospiraceac UCG-003*	0.21 ± 0.31	0.48 ± 0.71	0.027
					*Faecalibacterium*	6.62 ± 5.79	10.43 ± 7.25	0.029
					*Murdochiella*	0.0003 ± 0.0008	0.0006 ± 0.004	0.054

Values are mean ± standard deviation.

PSCI indicates post-stroke cognitive impairment; Non-PSCI indicates none of the post-stroke cognitive impairment.

**Table 3:** The logistic regression model compares the expression of gut Microbiome between the PSCI and the non-PSCI group.

Level	Variables	Model I		Model II		Model III	
Order	*Clostridiales*	2.56 (0.78–8.38)	0.119	2.08 (0.59–7.31)	0.254	2.28 (0.57–9.03)	0.253
	*Oscillospirales*	0.93 (0.88–0.99)	**0.020**	0.93 (0.87–0.99)	**0.046**	0.91 (0.83–1.00)	0.053
Family	*Bacteroidaceae*	1.02 (0.99–1.05)	0.084	1.02 (0.99–1.05)	0.279	1.03 (0.99–1.07)	0.167
	*Clostridiaceae*	2.56 (0.78–8.35)	0.120	2.06 (0.59–7.19)	0.259	2.28 (0.57–9.04)	0.242
	*Selenomonadaceae*	1.15 (0.85–1.56)	0.364	1.35 (0.97–1.87)	0.077	1.36 (0.93–1.99)	0.118
	*Prevotellaceae*	0.97 (0.94–0.99)	**0.039**	0.97 (0.93–1.01)	0.121	0.97 (0.93–1.01)	0.179
	*Ruminococcaceae*	0.92 (0.85–0.99)	**0.021**	0.91 (0.84–0.99)	**0.043**	0.89 (0.79–0.99)	**0.044**
Genus	*Bacteroides*	1.02 (0.99–1.05)	0.086	1.02 (0.99–1.05)	0.277	1.03 (0.99–1.07)	0.173
	*Prevotellaceae_NK3B31_group*	1.30 (0.85–1.99)	0.227	1.16 (0.67-2.00)	0.604	1.89 (0.82–4.34)	0.136
	*Clostridium_sensu_stricto_1*	2.46 (0.78–7.73)	0.124	2.00 (0.59–6.72)	0.265	2.22 (0.58–8.45)	0.244
	*Megamonas*	1.14 (0.85–1.53)	0.376	1.34 (0.97–1.84)	0.077	1.36 (0.93–1.99)	0.113
	*Enterobacter*	5.86 (0.89–38.54)	0.066	14.45 (1.61–130.6)	**0.017**	11.79 (1.54–90.04)	**0.017**
	*Prevotella*	0.97 (0.94–0.99)	**0.040**	0.97 (0.93–1.01)	0.140	0.97 (0.93–1.01)	0.173
	*Catenibacterium*	0.91 (0.54–1.52)	0.720	1.03 (0.56–1.90)	0.914	0.87 (0.14–5.52)	0.882
	*Coprococcus*	0.65 (0.22–1.94)	0.437	0.66 (0.18–2.39)	0.529	0.56 (0.08–3.90)	0.560
	*Roseburia*	0.79 (0.47–1.32)	0.362	0.87 (0.51–1.49)	0.620	0.74 (0.40–1.37)	0.333
	*Faecalibacterium*	0.91 (0.84–0.98)	**0.020**	0.91 (0.82–0.99)	**0.040**	0.90 (0.79–1.01)	0.339
	*Oscillibacter*	0.18 (0.03–1.03)	0.054	0.26 (0.05–1.38)	0.114	0.25 (0.03–2.49)	0.236
	*Oscillospiraceac_UCG_003*	0.26 (0.06–1.03)	0.054	0.29 (0.07–1.23)	0.093	0.29 (0.05–1.70)	0.171

Model I crude model.

Model II adjusted age and gender, education level.

Model III additional adjusted BMI, and mRS.

PSCI indicates post-stroke cognitive impairment; Non-PSCI indicates none of the post-stroke cognitive impairment.

### Plasma levels of inflammatory biomarkers between the two groups

Among the 88 patients who received inflammatory biomarker examination, including 27 PSCI patients and 61 non-PSCI patients, plasma exotoxin (*p* = 0.041) and IL-12p40 (*p* = 0.031) levels were significantly higher in the PSCI group than in the non-PSCI group (Table 4).

**Table 4:** Comparison of plasma levels of biomarkers between the PSCI and non-PSCI groups.

	Total (*N* = 88)	PSCI (*N* = 27)	Non-PSCI (*N* = 61)	*p*	P*
Eotaxin	87.3 ± 3.4	97.5 ± 6.1	82.5 ± 4.0	0.041	0.225
G-CSF	14.3 ± 1.5	13.3 ± 2.4	14.8 ± 2.0	0.646	0.974
IFNγ	8.4 ± 0.8	6.6 ± 1.0	9.2 ± 1.0	0.067	0.162
IL-10	1.7 ± 0.1	1.9 ± 0.2	1.6 ± 0.1	0.195	0.375
IL-12p40	0.5 ± 0.1	1.0 ± 0.3	0.3 ± 0.1	0.031	0.913
IL-12p70	3.1 ± 0.2	3.0 ± 0.4	3.2 ± 0.3	0.667	0.956
IL-13	1.4 ± 0.2	1.3 ± 0.2	1.5 ± 0.2	0.470	0.316
sCD40L	2527.1 ± 234.5	2527.6 ± 394.1	2526.9 ± 292.6	0.999	0.845
IL-17 A	2.4 ± 0.2	2.0 ± 0.2	2.5 ± 0.2	0.122	0.211
IL-1α	1.9 ± 0.7	0.8 ± 0.6	2.5 ± 1.1	0.172	0.451
IL-1β	1.6 ± 0.1	1.6 ± 0.1	1.6 ± 0.1	0.858	0.949
IL-2	1.2 ± 0.0	1.3 ± 0.1	1.1 ± 0.0	0.106	0.455
IL-4	0.5 ± 0.2	0.3 ± 0.3	0.6 ± 0.3	0.510	0.675
IL-6	0.7 ± 0.1	0.8 ± 0.2	0.7 ± 0.1	0.702	0.572
IL-8	4.2 ± 0.4	4.1 ± 0.9	4.2 ± 0.4	0.834	0.458
MCP-1	233.8 ± 12.0	219.8 ± 16.0	239.8 ± 15.7	0.376	0.326
TNFα	19.1 ± 1.0	19.7 ± 2.1	18.8 ± 1.2	0.695	0.746

Values are median ± standard error.

*Adjustment for age and sex.

PSCI indicates post-stroke cognitive impairment; Non-PSCI indicates none of the post-stroke cognitive impairment.

### Correlation between microbiota and inflammatory biomarkers

**Fig. 5:**
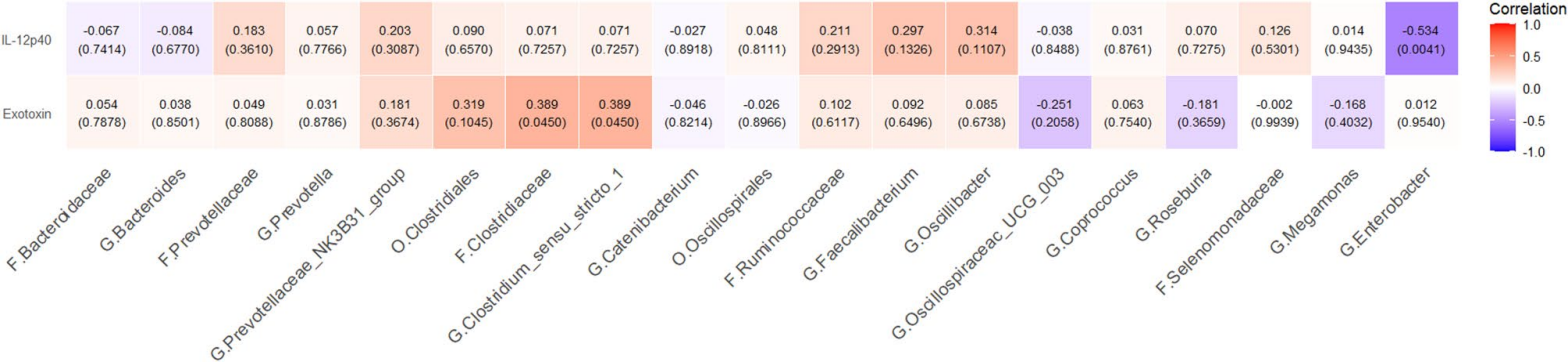
Correlation with microbiota and plasma inflammatory markers. Heatmap of Spearman rank correlation showing associations between microbiota and inflammatory markers exotoxin and IL-12p40; The values in the grid are the Spearman’s correlation coefficients, the *p*-values are in bracket, and the background color is red for positive correlation and blue for negative correlation. (PSCI, post-stroke cognitive impairment; non-PSCI, none of the post-stroke cognitive impairment; O, order; F, family; G, genus)

The pro-inflammatory marker exotoxin and IL-12p40, which exhibited significantly higher levels in the PSCI group, were selected to investigate their correlation with significant microbiota. The results revealed a significantly positive correlation between eotaxin and *Clostridiaceae* Family (rho = 0.389, *p* = 0.045) and *Clostridium_sensu_stricto_1* Genus (rho = 0.389, *p* = 0.045) (Fig. 5).

## Discussion

In this study, the findings highlight the association between specific fecal microbiota and the occurrence of cognitive impairment in ischemic stroke patients. While there was no significant difference in microbial abundance at the phylum level, there were notable differences at the family level, with increased abundance of *Bacteriodaceae*, *Clostridaceae*, and *Selenomonadeaceae* in the PSCI group, and *Prevotellaceae* and *Ruminococcaceae* in the non-PSCI group.

Our study used the MoCA to classify PSCI^30^ Previous studies have indicated that the MoCA demonstrates good reliability and validity in assessing mild cognitive impairment and post-stroke cognitive impairment^30–32^ In addition, the diagnosis of vascular cognitive impairment confirms that the patient’s cognitive level tends to decline after a stroke, with a decrease in the MoCA score of more than 2 points as the recommendation^33–36^ Grouping in our study combined the above suggestion. The PSCI group was defined as individuals whose single MoCA scale at 3-month or 1-year after stroke was less than 23, or a decrease of more than 2 points on the 1-year MoCA scale compared with the 3-month MoCA scale.

Previous research has shown alterations in gut microbiota composition following stroke. A review of 14 studies utilizing 16 S rDNA sequencing on collected fecal samples revealed 62 upregulated and 29 downregulated microbial taxa associated with clinical stroke^13^ However, limitations were identified, with few studies analyzing results by stroke subtypes and severity. Among these studies, three investigated the association between PSCI and gut microbiota. In one study involving 65 patients with acute ischemic stroke, those with PSCI had an increased abundance of *Fusobacterium*, *Bacteroides*, *Clostridium XlVa*, *Gemella*, and *Flavonifractor*, and a reduced abundance of *Oscillibacter*, *Ruminococcus*, *Gemmiger*, and *Coprococcus*^37^ Another study including 93 patients with ischemic stroke, where PSCI was assessed by MoCA scores 3 months after stroke onset, found an association with increased abundance of *Streptococcus*, *Klebsiella*, *Lactobacillus*, *Prevotella*, and *Veillonella* and decreased abundance of *Roseburia* and *Fusicatenibacter*^38^.

In a recent study where cognitive function was evaluated by MoCA 3 months post-stroke onset, patients with PSCI had higher levels of gut *Enterobacteriaceae* and plasma IL-6, Il-1β compared to those without PSCI^39^ Additionally, a meta-analysis including 12 studies, all conducted in China, revealed that patients with PSCI had an increased abundance of *Proteobacteria* at the phylum level; *Bacteroidaceae*, *Lachnospiraceae*, and *Veillonellaceae* at the family level; and *Bacteroides*, *Clostridium XIVa*, and *Parabacteroides* at the genus level. Conversely, compared to controls, they displayed decreased abundance of Enterobacteriaceae at the family level and Prevotella and Ruminococcus at the genus level^40^ While our study did not observe significant differences at the phylum level, we did find an increased abundance of *Bacteroidaceae* and a decreased abundance of *Ruminococcaceae*,* Prevotella* in PSCI patients compared to non-PSCI patients.

Some animal studies have reported an increased abundance of *Bacteroidetes* following cerebral ischemia,^41,42^ but findings from clinical studies have been inconsistent^43,44^ In our study, we observed a similar abundance of *Bacteroidetes* but a higher abundance of *Bacteroidaceae* in the PSCI patients, consistent with findings from other studies^38,45^ *Prevotella*, a genus within the phylum *Bacteroidetes*, has been known as an important pathobiont in various diseases due to its role in promoting chronic inflammation^46^ However, some *Prevotella* species, such as *Prevotella histicola*, have shown potential in mitigating neurological impairment. A recent study found that transplantation of *Prevotella histicola* to rats with vascular dementia could alleviate disease progression^47^.

*Ruminococcaceae* has been known to play a role in the regulation of inflammation and related to AD^48,49^ A recent study with a 2-year follow-up in 87 patients with MCI found that a decreased abundance of *Ruminococcaceae*, correlated with an increase in P-tau181, was significantly associated with the progression of MCI^49^ Our study showed a decreased abundance of *Ruminococcaceae* associated with cognitive impairment after stroke. Additionally, we also found a reduced abundance of *Faecalibacterium* and *Oscillibacter*. A monkey study observed decreased relative abundance levels of *Faecalibacterium*, *Oscillospira*, and *Lactobacillus* after cerebral infarction^42^ *Faecalibacterium* and *Oscillospira* have been considered the primary source of butyrate, which plays a crucial role in maintaining the integrity of the intestinal barrier and inhibiting the production of pro-inflammatory cytokines^50^.

Acute stroke can trigger localized inflammation in the injured brain region, leading to global brain inflammation through disruption of the blood-brain barrier, microglial activation, and increased secretion of soluble inflammatory mediators such as C-reactive protein, IL-6, IL-12, and TNF-α. These series of inflammation markers are associated with cognitive decline and stroke recurrence^51–55^ Our study found that the plasma levels of exotoxin and IL-12p40 were significantly higher in the PSCI group than in the non-PSCI group. However, after adjusting for age and sex, this difference became less significant. Eotaxin, recognized as an endogenous cognitive-deteriorating chemokine, has been associated with cognitive deficits^56^ In a multi-ethnic cohort study, higher eotaxin levels were associated with decreased cognitive performance in 1179 stroke-free participants^57^ IL-12p40, a subunit shared by IL-12 and IL-23, is produced in response to various inflammatory stimuli^58^ Some studies have demonstrated that elevated IL-12p40 levels are associated with an increased risk of cognitive impairment and intracerebral hemorrhage^59,60^ The significant differences in exotoxin and IL-12p40 levels disappeared after adjustment for age and sex, suggesting a more age-related influence. Additionally, the lack of plasma measurements during the acute phase of stroke and the fact that most patients in the study had mild strokes and were on antithrombotic medications could explain these findings. Therefore, these biomarkers may not be sensitive enough to differentiate the mild inflammatory response contributing to cognitive impairment post-stroke.

The advantage of this study is that our PSCI definition was based not only on a single MoCA score but also on MoCA changes. Rather than using only one test for current status, we assessed MoCA both at the 3-month and 1-year after stroke, which allowed us to ensure stroke patients’ cognitive status deterioration. However, this study has several limitations. First, the majority of the recruited patients had mild strokes and mainly small vessel disease, which may not fully represent the diverse stroke patient population in the real world. Second, the one-year period after stroke might not have been long enough to detect the occurrence of cognitive impairment or dementia accurately. Third, there were significant differences in some variables, including age and education, between patients with and without PSCI. Despite efforts to perform age- and sex-matched comparisons, adjusting for several known or unknown characteristics, such as dietary changes, infections, and antibiotic exposure during the 3-month to 1-year period was impossible. These limitations should be considered when interpreting the results of the study.

In conclusion, our study revealed associations between specific gut microbiota, particularly *Clostridiaceae* and *Prevotellaceae*, and PSCI. Notably, these gut microbiotas were also found to correlate with elevated levels of the pro-inflammatory marker eotaxin, suggesting an inflammatory pathway that may contribute to the development of cognitive decline following ischemic stroke. These findings offer valuable insights into the potential role of the gut-brain axis in post-stroke neuroinflammation and cognitive dysfunction. Given the modifiable nature of the gut microbiome, these results open up promising avenues for developing microbiome-based therapeutic strategies to prevent or mitigate PSCI. Further validation of these findings in more extensive, longitudinal clinical cohorts is warranted to confirm their clinical relevance, explore the underlying mechanisms, and assess the potential for microbiota-targeted interventions in managing cognitive outcomes in stroke survivors.

## Data Availability

All data from this article are being held within Taipei Medical University and are available from the corresponding author upon reasonable request. The nucleotide sequence datasets generated and/or analysed during the current study are available in the NCBI genome database under BioProject accession number PRJNA1246515 https://www.ncbi.nlm.nih.gov/sra/PRJNA1246515.
